# Supplementary material for: Interventions to increase early infant diagnosis of HIV infection: A systematic review and meta-analysis
Source: PLoS One. 2022 Feb 25;17(2):e0258863. doi: 10.1371/journal.pone.0258863 (PMC8880648; doi:10.1371/journal.pone.0258863)
Supplement: S1 Table — (DOCX) [file pone.0258863.s001.docx]

S1. Database search strategies

| PubMed | EMBASE | CINAHL | Web of Science | PsycINFO |
| --- | --- | --- | --- | --- |
| (Intervention OR “nevirapine prophylaxis” OR zidovudine OR antiretroviral OR tracking OR “conditional cash transfer” OR follow up OR accompaniment OR referral OR immunization OR Integration OR “mother-to-mother” support OR “mentor mothers” OR Peer support OR cognitive behavior therapy OR Mobile OR SMS OR “methods”[mesh] OR “nevirapine”[mesh] OR “zidovudine”[mesh] OR “vaccination”[mesh] OR “cognitive behavioral therapy”[mesh])  AND  (HIV OR AIDS OR “acquired immunodeficiency” OR “HIV”[MeSH] OR “acquired immunodeficiency syndrome”[mesh])  AND  (Pediatric diagnosis OR Early infant diagnosis OR Point of care diagnosis OR infant testing OR HIV infant testing OR “point-of-care testing”[mesh])  AND  (mother to child transmission OR maternal to child transmission OR adult to child transmission OR vertical transmission OR vertical disease transmission OR PMTCT OR EMTCT OR Breastfeeding OR "Infectious Disease Transmission, Vertical/prevention and control"[Mesh])  AND  (Uptake OR adherence OR follow up OR retention OR attrition OR Compliance OR Recruit OR "patient compliance"[MeSH] OR “compliance”[mesh]) | (hiv OR aids OR 'acquired immunodeficiency' OR 'human immunodeficiency virus'/exp) AND ('pediatric diagnosis' OR 'early infant diagnosis' OR 'point of care diagnosis' OR 'infant testing' OR 'hiv infant testing' OR 'point of care testing'/exp) AND ('mother to child transmission' OR 'maternal to child transmission' OR 'adult to child transmission' OR 'vertical transmission' OR 'vertical disease transmission' OR pmtct OR emtct OR breastfeeding OR 'vertical transmission'/exp) AND (uptake OR adherence OR 'follow up' OR retention OR attrition OR compliance OR recruit OR use OR 'patient compliance'/exp OR 'follow up'/exp) AND (intervention OR 'nevirapine prophylaxis' OR zidovudine OR antiretroviral OR tracking OR 'conditional cash transfer' OR 'follow up' OR accompaniment OR referral OR immunization OR integration OR 'mother-to-mother support' OR ‘mentor mothers’ OR ‘peer support’ OR 'cognitive behavior therapy' OR mobile OR sms OR 'intervention'/exp OR 'zidovudine'/exp OR 'nevirapine'/exp OR 'referral'/exp OR 'immunization'/exp OR 'integration'/exp OR 'cognitive behavioral therapy'/exp OR 'mobile application'/exp) | (intervention OR “nevirapine prophylaxis” OR zidovudine OR antiretroviral OR tracking OR “conditional cash transfer” OR “follow up” OR accompaniment OR referral OR immunization OR integration OR “mother-to-mother” support” OR “mentor mothers” OR “peer support” OR “cognitive behavior therapy” OR mobile OR sms OR  (MH “zidovudine”) OR (MH “nevirapine”) OR (MH “Immunization”) OR (MH “Referral and Consultation”) OR (MH “Peer Counseling”) OR (MH “Mobile Applications”) OR (MH “Cognitive Therapy”))  AND  (uptake OR adherence OR “follow up” OR retention OR attrition OR compliance OR recruit OR use OR  (MH “Patient Compliance”) OR (MH “After Care”))  AND  (“mother to child transmission” OR “maternal to child transmission” OR “adult to child transmission” OR “vertical transmission” OR “vertical disease transmission” OR PMTCT OR EMTCT OR breastfeeding OR  (MH “Disease Transmission, Vertical))  AND  (“pediatric diagnosis” OR “early infant diagnosis” OR “point of care diagnosis” OR “infant testing” OR “HIV infant testing” OR (MH Point-of-Care Testing”))  AND  (HIV OR AIDS OR “acquired immunodeficiency” OR  (MH “Human Immunodeficiency Virus)) | TS=(intervention OR “nevirapine prophylaxis” OR zidovudine OR antiretroviral OR tracking OR “conditional cash transfer” OR “follow up” OR accompaniment OR referral OR immunization OR integration OR “mother-to-mother support” OR “mentor mothers” OR “peer support” OR “cognitive behavior therapy” OR mobile OR sms)  AND  TS=(uptake OR adherence OR “follow up” OR retention OR attrition OR compliance OR recruit OR use)  AND  TS=(“mother to child transmission” OR “maternal to child transmission” OR “adult to child transmission” OR “vertical transmission” OR “vertical disease transmission” OR PMTCT OR EMTCT OR breastfeeding)  AND  TS=(“pediatric diagnosis” OR “early infant diagnosis” OR “point of care diagnosis” OR “infant testing” OR “HIV infant testing”)  AND  TS=( HIV OR AIDS OR “acquired immunodeficiency”) | (intervention OR “nevirapine prophylaxis” OR zidovudine OR antiretroviral OR tracking OR “conditional cash transfer” OR “follow up” OR accompaniment OR referral OR immunization OR integration OR “mother-to-mother” support” OR “mentor mothers” OR “peer support” OR “cognitive behavior therapy” OR mobile OR sms OR  DE “Peer Counseling” OR DE “mobile devices” OR DE "Cognitive Behavior Therapy")  AND  (uptake OR adherence OR “follow up” OR retention OR attrition OR compliance OR recruit OR use OR  DE “Treatment Compliance” OR DE “Experimental Attrition”)  AND  (“mother to child transmission” OR “maternal to child transmission” OR “adult to child transmission” OR “vertical transmission” OR “vertical disease transmission” OR PMTCT OR EMTCT OR breastfeeding)  AND  (“pediatric diagnosis” OR “early infant diagnosis” OR “point of care diagnosis” OR “infant testing” OR “HIV infant testing”)  AND  (HIV OR AIDS OR “acquired immunodeficiency” OR  DE “HIV” OR DE “HIV Testing”) |
