# Supplementary material for: Interventions to increase early infant diagnosis of HIV infection: A systematic review and meta-analysis
Source: PLoS One. 2022 Feb 25;17(2):e0258863. doi: 10.1371/journal.pone.0258863 (PMC8880648; doi:10.1371/journal.pone.0258863)
Supplement: S2 Table — (DOCX) [file pone.0258863.s002.docx]

Appendix 2: Table of excluded studies

| Serial No. | Study ID | Reason for exclusion |  | Serial No. | Study ID | Reason for exclusion |
| --- | --- | --- | --- | --- | --- | --- |
| 1. | Ahmed 2015 | Ineligible patient population |  | 29. | Kiyaga 2018 | Ineligible study design |
| 2. | Ahoua 2010 | No EID interventions |  | 30. | Kiyaga 2018a | Ineligible study design |
| 3. | Aliyu 2013 | Study protocol |  | 31. | Mazanderani 2018 | Ineligible study design |
| 4. | Aliyu 2014 | Ineligible study design |  | 32. | McCollum 2012 | Data in unusable format |
| 5. | Aliyu 2016 A | Available only as abstract |  | 33. | Moses 2008 | No EID interventions |
| 6. | Anaba 2017 | Ineligible outcomes |  | 34. | Mudany 2015 | Data in unusable format |
| 7. | Anaba 2019 | Ineligible outcomes |  | 35. | Namukwaya 2015 | Data in unusable format |
| 8. | Awiti 2016 | Study protocol |  | 36. | Ndondoki 2013 | Ineligible study design |
| 9. | Beyene 2017 | Available only as abstract |  | 37. | Onwubiko 2019 | Available only as abstract |
| 10. | Bianchi 2018 | Ineligible study design |  | 38. | Oyeledun 2017 | Ineligible outcomes |
| 11. | Binagwaho 2013 | No EID interventions |  | 39. | Peltzer 2011 | Study protocol |
| 12. | Ciampa 2011 | Data in unusable format |  | 40 | Pharr 2013 | Data in unusable format |
| 13. | Ciampa 2012 | Data in unusable format |  | 41. | Rawizza 2015 | Ineligible study design |
| 14. | Doherty 2009 | Ineligible patient population |  | 42. | Reimers 2016 | Study protocol |
| 15. | Drake 2017 | Study protocol |  | 43. | Sam Agudu 2014 | Available only as abstract |
| 16. | Etoori 2018 | Ineligible study design |  | 44. | Sando 2014 | Study protocol |
| 17. | Ezeanolue 2013 | Study protocol |  | 45. | Sau 2016 | Ineligible study design |
| 18. | Feinstein 2015 | No EID interventions |  | 46. | Tejiokem 2011 | No EID interventions |
| 19 | Finocchario Kessler 2015 | Ineligible outcomes |  | 47. | Turan 2012 | Study protocol |
| 20. | Finocchario Kessler 2015a | Study protocol |  | 48. | Turan 2012 | Ineligible study design |
| 21. | Finocchario Kessler 2019 | Study protocol |  | 49. | Turan 2015 | Data in unusable format |
| 22. | Gamell 2015 | Available only as abstract |  | 50. | Wiegert 2014 | Ineligible study design |
| 23. | Gamell 2016 | Ineligible patient population |  | 51. | Woelk 2016 | Study protocol |
| 24. | Gamell 2016a | Ineligible patient population |  |  |  |  |
| 25. | Geelhoed 2013 | Ineligible outcomes |  |  |  |  |
| 26. | Goodson 2003 | Data in unusable format |  |  |  |  |
| 27. | **Kim 2012** | Data in unusable format |  |  |  |  |
| 28. | Kiyaga 2015 | Ineligible study design |  |  |  |  |
