# Supplementary material for: Interventions to increase early infant diagnosis of HIV infection: A systematic review and meta-analysis
Source: PLoS One. 2022 Feb 25;17(2):e0258863. doi: 10.1371/journal.pone.0258863 (PMC8880648; doi:10.1371/journal.pone.0258863)
Supplement: S3 Table — (DOCX) [file pone.0258863.s003.docx]

**Appendix 3: Characteristics of included studies**

| eHealth Interventions | |
| --- | --- |
| Coleman 2017 | |
| Methods | Retrospective cohort study |
| Participants | Pregnant women living with HIV who are over the age of 18 years, be with access to a cell phone and delivered in one of the facilities |
| Interventions | Intervention:  MAMA SMSs: on healthy eating, reminders of ANC/PNC appointments, psycho-social support, delivery planning), PCR testing reminders, adherence to ART  Control:  Usual care |
| Outcomes | DNA PCR test performed at 4-8 weeks  Identification of HIV-infected infants |
| Identification | Country: South Africa  Authors name: Jesse Coleman  Email: [jesse.coleman@ki.se](mailto:jesse.coleman@ki.se) |
| Funding | This study was funded in part by Johnson & Johnson; Vodacom Foundation; United States Agency for International Development [grant number AID-674-A-12-00004]; the European Union Horizon 2020 Sci-GaIA project [grant number 654237]; and the Swedish Foundation for Clinical Pharmacology and Pharmacotherapy |
| Kassaye 2016 | |
| Methods | Cluster randomized controlled trial |
| Participants | Eligible pregnant women living with HIV, presenting for antenatal care less than 32 weeks of gestational age, were not currently receiving anti-retroviral therapy, were planning to remain in the area for the duration of the study period, and agreed to follow-up of their infants until 6 weeks following delivery |
| Interventions | mHealth platform  SMS text messages (3-6/ week) in the local language covering: PMTCT services; motivational messages and male-partner involvement; warning signs and nutrition.  Control  No description was provided: |
| Outcomes | DNA PCR Test performed at 4-6 weeks |
| Identification | Country: Kenya  Authors name: Seble G. Kassaye  Email: [sgk23@georgetown.edu](mailto:sgk23@georgetown.edu) |
| Funding | World Health Organization through a grant to the Elizabeth Glaser Pediatric AIDS Foundation and by the Elizabeth Glaser Pediatric AIDS Foundation |
| Odeny 2014 | |
| Methods | Randomized controlled trial |
| Participants | Pregnant women living with HIV >18 years old between 28 weeks gestation and delivery, enrolled in PMTCT. Other eligibility criteria are plans to remain in the study area, had access to a mobile phone, and reported ability to read or had someone who read short message service (SMS) on their behalf. Women who shared phones were eligible only if they had disclosed their HIV status to the person with whom the phone was shared |
| Interventions | Intervention:  Individually tailored, theory-based two-way SMS. The intervention consisted of 14 messages; 8 during pregnancy, and weekly for the first 6 weeks after delivery.  Control:  Standard of care; no SMS |
| Outcomes | DNA PCR Test performed at 4-8 weeks  Identification of HIV-infected infant |
| Identification | Country: Kenya  Authors name: Thomas A. Odeny  Email: odeny@uw.edu. |
| Funding | National Institutes of Health Office of the Director, Fogarty International Center |

| eHealth Interventions (Contd.) | |
| --- | --- |
| Odeny 2019 | |
| Methods | Stepped-wedge Cluster randomized control trial |
| Participants | Pregnant women living with HIV enrolled in the PMTCT program at target health facilities. Inclusion criteria include women without mobile phones. Women were offered the opportunity to receive text messages if they were 18 years old or emancipated minors and between 28 weeks’ gestation and delivery. Similar criteria were used to abstract data for women at control facilities |
| Interventions | Intervention:  Individually tailored, theory-based two-way SMS. The intervention consisted of 14 messages; 8 during pregnancy SMS system received up to 14 text messages during pregnancy and after delivery, and weekly for the first 6 weeks after delivery  Control:  Usual care |
| Outcomes | DNA PCR Test performed at 4-8 weeks |
| Identification | Country: Kenya  Authors name: Thomas A. Odeny  Email: [odeny@uw.edu](mailto:odeny@uw.edu) |
| Funding | United Nations Foundation under the Innovation Working Group catalytic funding initiative to strengthen evaluation form Health scale and sustainability (UNF-13-545awardedtoTAO). |
| Sarna 2019 | |
| Methods | Randomized controlled trial |
| Participants | Pregnant women living with HIV who were between 14 and 36 weeks of gestation, aged≥16 years, residing in Kisumu and planning to stay there for the next 12 months, willing and able to provide consent, and who had access to a cell phone (owned or shared) to participate in the study. Participants could be ART naïve or experienced (they were currently on ART or had received nevirapine for a previous pregnancy) |
| Interventions | Intervention:  A structured, counselor-delivered, tailored cell phone counseling; one-on-one individualized counseling, delivered via cell phone by 5 trained counselors based at a central study office.  Control:  Usual care |
| Outcomes | DNA PCR Test performed at 4-8 weeks  Identification of HIV-infected infant |
| Identification | Country: Kenya  Authors name: Avina Sarna  Email: [asarna@popcouncil.org](mailto:asarna@popcouncil.org) |
| Funding | U.S. President's Emergency Plan for AIDS Relief and the U.S.Agency for International Development (USAID) via HIVCore, a Task Order funded by USAID under the Project SEARCH |
| Schwartz 2015 | |
| Methods | Before and After study |
| Participants | Pregnant women living with HIV attending antenatal care from May–July 2013, ≥36 weeks’ gestation. Additional eligibility criteria included owning a cell phone, being able to read text messages in English, and willingness to receive text messages and calls from a case manager (CM). Both women already on HAART at time of pregnancy and women not yet HAART-initiated at time of pregnancy were eligible for this study. |
| Interventions | Intervention:  Weekly SMS messages to the participants’ cell phones until 6 weeks postpartum (or up to 8 weeks if the participant did not return for the 6-week clinic visit), and one pre-delivery and two post-delivery telephone calls.  Control:  A retrospective file review of 50 HIV-infected, pregnant women attending ANC at WHWC during the period immediately preceding the intervention (February–May, 2013). |
| Outcomes | DNA PCR Test performed at 4-8 weeks  Identification of HIV-infected infant |
| Identification | Country: South Africa  Authors name: Sheree Schwartz  Email: [heree_schwartz@med.unc.edu](mailto:heree_schwartz@med.unc.edu) |
| Funding | This study was funded by the United States Agency for International Development (USAID) under award number AID6741200033. Sheree Schwartz received support for this work from the UJMT Fogarty Grant, supported by the Fogarty International Center of the National Institutes of Health under Award Number R25TW009340. SS, KC, AVR, JB & AP conceived of the study; |

| Health systems improvement interventions | |
| --- | --- |
| Finocchario Kessler 2014 | |
| Methods | Before and After study |
| Participants | This study did not require active recruitment because all mother/guardian–infant pairs presenting for EID care were eligible to utilize the HITSystem. |
| Interventions | Intervention:  Internet-based program, which captures clinical data and uses the infant’s birth date to trigger electronic alerts. Text messages are sent to mothers’ mobile phones when test results are available, medical treatment is indicated  Control:  Not applicable |
| Outcomes | DNA PCR Test performed at 4-8 weeks  Identification of HIV-infected infant  Turnaround time of EID result to caregiver  Turnaround time of result availability to mother  Initiation of anti-retroviral therapy by an HIV-positive infant |
| Identification | Country: Kenya  Authors name: Sarah Finocchario-Kessle  Email: [Skessler2@kumc.edu](mailto:Skessler2@kumc.edu) |
| Funding | Global Health Innovations and Health Empowering Humanity through private donations, and the National Institutes of Child Health and Development, R01HD076673 |
| Finocchario Kessler 2018 | |
| Methods | Cluster randomized trial |
| Participants | Women living with HIV aged 18 years or older with an HIV-exposed infant presenting for their first early infant diagnosis appointment through the maternal and child health department; and presented for early infant diagnosis care when their infant was younger than 24 weeks |
| Interventions | Intervention:  HIV Infant Tracking System (HITSystem): HITSystem  Control:  Standard of care: Standard o f care |
| Outcomes | DNA PCR Test performed at 4-8 weeks  Identification of HIV-infected infant  Turnaround time of EID result to caregiver  Turnaround time of result availability to mother  Initiation of anti-retroviral therapy by an HIV-positive infant |
| Identification | Country: Kenya  Authors name: Sarah Finocchario-Kessle  Email: [Skessler2@kumc.edu](mailto:Skessler2@kumc.edu) |
| Funding | National Institute of Child Health and Human Development |
| Gupta 2016 | |
| Methods | Before and After study |
| Participants | HIV field level staff who follow-up of HIV-exposed babies |
| Interventions | Intervention:  ‘EID Follow-up system: a web-based tool which generated automated SMS and e-mails for reminding the field level staff  Control:  Standard of care |
| Outcomes | DNA PCR Test performed at 4-8 weeks |
| Identification | Country: India  Authors name: Asha S. Hegde  Email: [drashahegde@gmail.com](mailto:drashahegde@gmail.com) |
| Funding | None |
| Herlily 2015 | |
| Methods | Quasi-experimental (Before and After) study |
| Participants | All pregnant women living with HIV not on ART at the time of first ANC |
| Interventions | Intervention:  Our intervention had 3 components: (1) training of 132 ANC providers, (2) establishment of laboratory courier system to expedite CD4 results, and (3) follow-up of mother–infant pairs by 82 community-based lay counselors.  Control:  Usual care |
| Outcomes | DNA PCR Test performed at 4-8 weeks |
| Identification | Country: South Africa  Author: Julie M. Herlihy, MD, MPH,  Email: [herlihyj@gmail.com](mailto:herlihyj@gmail.com) |
| Funding | Supported by cooperative agreement number 5U2GPS001418 from the Centers of Disease Control and Prevention, with the support of the Coordinating Office of Global Health |

| Service integration interventions | |
| --- | --- |
| Aliyu 2016 | |
| Methods | Cluster randomized trial |
| Participants | Pregnant women living with HIV and their children |
| Interventions | Intervention:  Integrated package of PMTCT services: point-of-care CD4 cell count or percentage testing; decentralised PMTCT tasks to trained midwives (task shifting), integrated mother and infant care services, male partner participation, and community involvement  Control:  Usual care |
| Outcomes | DNA PCR Test performed at 4-8 weeks  Identification of HIV-infected infant |
| Identification | Country: Nigeria  Authors name: Muktar H Aliyu  Email: [muktar.aliyu@vanderbilt.edu](mailto:muktar.aliyu@vanderbilt.edu) |
| Funding | Eunice Kennedy Shriver National Institute of Child Health & Human Development of the National Institutes of Health (R01HD075075), National Institutes of Health-funded Tennessee CFAR (P30 AI110527), |
| Washington 2015 | |
| Methods | Cluster randomized control trial |
| Participants | Pregnant women living with HIV 18 years and older, not previously enrolled in HIV care, and followed each mother for one year after enrollment. Infants were followed until nine months after delivery. |
| Interventions | Intervention:  Integration of ANC, PMTCT, and HIV care including enrollment in HIV care, management of HIV disease, management of opportunistic infections and HAART if eligible. The same clinician provided all antenatal and postpartum services including early infant diagnosis until a definitive pediatric HIV diagnosis was obtained or the child reached 18 months of age.  Control:  Usual care; stand-alone ANC and PMTCT clinics. |
| Outcomes | DNA PCR Test performed at 4-8 weeks  Identification of HIV-infected infant |
| Identification | Country: Kenya  Authors name: Sierra Washington  Email: [sierra.washington@gmail.com](mailto:sierra.washington@gmail.com) |
| Funding | The President's Emergency Plan for AIDS Relief (PEPFAR)/U.S. Centers for Disease Control and Prevention (CDC). The study was funded under the CDC cooperative agreement number 5U2GPS001913-02. |

| Behavioral interventions | |
| --- | --- |
| Igumbor 2019 | |
| Methods | Before and After |
| Participants | Women attending PMTCT clinics in Uganda between January 2011 and March 2014 |
| Interventions | Intervention:  The standard of care for PMTCT as well as the psychosocial support  Control:  Uganda usual standard of care for PMTCT services, the general PMTCT service package and family support groups |
| Outcomes | DNA PCR Test performed at 4-8 weeks |
| Identification | Country:  Authors name:  Email: |
| Funding | Not stated |
| Liu 2019 | |
| Methods | Randomized control trial |
| Participants | Pregnant women living with HIV attending ANC at 1 of the 3 participating study facilities, not previously participated in a conditional cash transfer (CCT) programme administered by the implementing non-governmental organization (NGO). |
| Interventions | Intervention: Conditional cash transfers during pregnancy through 10 weeks after birth for achieving specific milestones. One transfer per milestone achieved. Milestone 1: enrolled in the CCT programme to receive the following transfers Part 1: 1000 Naira (~US$3) + 300 Naira (~US$1) of mobile phone credits Part 2: 6000 Naira (~US$20). Milestone 2: Deliver at the facility where enrolled to receive 0 Naira (~US$70). Milestone 3: obtain an EID test (only eligible to women who delivered at the facility) to receive 6000 Naira (~US$20).  Control:  Usual care |
| Outcomes | DNA PCR Test performed at 4-8 weeks |
| Identification | Country: Nigeria  Authors name: Jenny X. Liu  Email: [Jenny.Liu2@ucsf.edu](mailto:Jenny.Liu2@ucsf.edu) |
| Funding | Bill & Melinda Gates Foundation [OPP1111515] |
| Sam-Agudu 2017 | |
| Methods | Prospective cohort study |
| Participants | Women living with HIV and their HIV-exposed infants, whether they are ART-naive and ART-experienced women. Women of all gestational ages making at least 1 antenatal visit were eligible for recruitment. |
| Interventions | Intervention:  Mentor-mother support: Use of Mentor Mothers (MMs), who are HIV-positive women who have successfully completed the PMTCT cascade and are trained to support less-experienced PMTCT clients  Control:  Usual care |
| Outcomes | DNA PCR Test performed at 4-8 weeks  Identification of HIV-infected infant |
| Identification | Country: Nigeria  Authors name: Nadia A. Sam-Agudu  Email: [nsamagudu@ihvnigeria.org](mailto:nsamagudu@ihvnigeria.org) |
| Funding | The MoMent Nigeria study was funded by the World Health Organization through an award for the Integrating and Scaling up PMTCT through Implementation research (INSPIRE) initiative from Global Affairs Canada. |
| Weiss 2014 | |
| Methods | Cluster randomized controlled trial |
| Participants | Pregnant women who had completed HIV counselling and testing (HCT) and were 24 to 30 weeks’ pregnant and ≥18 years of age |
| Interventions | Intervention:  Male partner involvement using The PartnerPlus intervention (cognitive–behavioral HIV risk reduction intervention) addressed HIV, safer sex, sexual negotiation, and PMTCT issues. The intervention consisted of 4 successive weekly sessions of 90 to 120 minutes each.  Control:  Standard of antenatal care  Identification of HIV-infected infant |
| Outcomes | DNA PCR Test performed at 4-8 weeks |
| Identification | Country: South Africa  Author: D. L. Jones  Email: [djones@med.miami.edu](mailto:djones@med.miami.edu). |
| Funding | This study was collaboratively funded by the National Institute of Allergy and Infectious Diseases (5P30AI073961-S2 Supplement grant to the University of Miami CFAR from a collaboration between NIAID and PEPFAR). |
