# Supplementary material for: Interventions to increase early infant diagnosis of HIV infection: A systematic review and meta-analysis
Source: PLoS One. 2022 Feb 25;17(2):e0258863. doi: 10.1371/journal.pone.0258863 (PMC8880648; doi:10.1371/journal.pone.0258863)
Supplement: S4 Table — (DOCX) [file pone.0258863.s004.docx]

**S4: Quality of evidence for ehealth interventions vs. usual care for randomized controlled trials**

| **Summary of findings:** | | | | | | |
| --- | --- | --- | --- | --- | --- | --- |
| **eHealth Interventions (Randomized studies) compared to Usual care for uptake of early infant diagnosis of HIV infection** | | | | | | |
| **Patient or population**: uptake of early infant diagnosis of HIV infection  **Setting**: HIV prevalent settings  **Intervention**: eHealth Interventions (Randomized studies)  **Comparison**: Usual care | | | | | | |
| Outcomes | **Anticipated absolute effects^*^** (95% CI) | | Relative effect (95% CI) | № of participants  (studies) | Certainty of the evidence (GRADE) | Comments |
|  | **Risk with Usual care** | **Risk with eHealth Interventions (Randomized studies)** |  |  |  |  |
| Uptake of early infant diagnosis at 4-8 weeks of age | 855 per 1,000 | 896 per 1,000 (875 to 914) | **OR 1.46** (1.18 to 1.80) | 3473 (4 RCTs) | ⨁⨁⨁◯ MODERATE ^a^ |  |
| Identification of HIV-infected infants | 15 per 1,000 | 21 per 1,000 (9 to 51) | **OR 1.41** (0.57 to 3.52) | 1049 (3 RCTs) | ⨁⨁⨁◯ MODERATE ^b^ |  |
| Turnaround time of EID result to caregiver - not measured | - | - | - | - | - |  |
| Turnaround time of EID result to mother - not measured | - | - | - | - | - |  |
| Initiation of anti-retroviral therapy by an HIV-positive infant - not measured | - | - | - | - | - |  |
| ***The risk in the intervention group** (and its 95% confidence interval) is based on the assumed risk in the comparison group and the **relative effect** of the intervention (and its 95% CI).   **CI:** Confidence interval; **OR:** Odds ratio | | | | | | |
| **GRADE Working Group grades of evidence** **High certainty:** We are very confident that the true effect lies close to that of the estimate of the effect **Moderate certainty:** We are moderately confident in the effect estimate: The true effect is likely to be close to the estimate of the effect, but there is a possibility that it is substantially different **Low certainty:** Our confidence in the effect estimate is limited: The true effect may be substantially different from the estimate of the effect **Very low certainty:** We have very little confidence in the effect estimate: The true effect is likely to be substantially different from the estimate of effect | | | | | | |

#### Explanations

a. Statistical moderate heterogeneity of 56%; Downgrade by 1

b. Downgraded by 1 for unmet optimal information size and wide confidence
