# Supplementary material for: Interventions to increase early infant diagnosis of HIV infection: A systematic review and meta-analysis
Source: PLoS One. 2022 Feb 25;17(2):e0258863. doi: 10.1371/journal.pone.0258863 (PMC8880648; doi:10.1371/journal.pone.0258863)
Supplement: S6 Table — (DOCX) [file pone.0258863.s006.docx]

**S6: Quality of evidence for interventions to integrate EID services vs. usual care**

| **Summary of findings:** | | | | | | |
| --- | --- | --- | --- | --- | --- | --- |
| **Integration of EID of HIV to other services compared to Usual care for uptake of early infant diagnosis of HIV infection** | | | | | | |
| **Patient or population**: uptake of early infant diagnosis of HIV infection  **Setting**:  **Intervention**: Integration of EID of HIV to other services  **Comparison**: Usual care | | | | | | |
| Outcomes | **Anticipated absolute effects^*^** (95% CI) | | Relative effect (95% CI) | № of participants  (studies) | Certainty of the evidence (GRADE) | Comments |
|  | **Risk with Usual care** | **Risk with Integration of EID of HIV to other services** |  |  |  |  |
| Uptake of early infant diagnosis at 4-8 weeks of age | 158 per 1,000 | 362 per 1,000 (310 to 418) | **OR 3.02** (2.39 to 3.82) | 1482 (2 RCTs) | ⨁◯◯◯ VERY LOW ^a,b,c^ |  |
| Identification of HIV-infected infants | 99 per 1,000 | 34 per 1,000 (14 to 78) | **OR 0.32** (0.13 to 0.77) | 389 (2 RCTs) | ⨁◯◯◯ VERY LOW ^a,b,c^ |  |
| Turnaround time of EID result to caregiver - not measured | - | - | - | - | - |  |
| Turnaround time of EID result to mother - not measured | - | - | - | - | - |  |
| Initiation of anti-retroviral therapy by an HIV-positive infant - not measured | - | - | - | - | - |  |
| ***The risk in the intervention group** (and its 95% confidence interval) is based on the assumed risk in the comparison group and the **relative effect** of the intervention (and its 95% CI).   **CI:** Confidence interval; **OR:** Odds ratio | | | | | | |
| **GRADE Working Group grades of evidence** **High certainty:** We are very confident that the true effect lies close to that of the estimate of the effect **Moderate certainty:** We are moderately confident in the effect estimate: The true effect is likely to be close to the estimate of the effect, but there is a possibility that it is substantially different **Low certainty:** Our confidence in the effect estimate is limited: The true effect may be substantially different from the estimate of the effect **Very low certainty:** We have very little confidence in the effect estimate: The true effect is likely to be substantially different from the estimate of effect | | | | | | |

#### Explanations

a. Downgraded by 1 because of risk of attrition bias in one of the studies (Washington 2015)

b. Downgraded by 1 because of substantial statistical heterogeneity

c. Downgraded by 1 because of small sample size
