# Supplementary material for: Interventions to increase early infant diagnosis of HIV infection: A systematic review and meta-analysis
Source: PLoS One. 2022 Feb 25;17(2):e0258863. doi: 10.1371/journal.pone.0258863 (PMC8880648; doi:10.1371/journal.pone.0258863)
Supplement: Attachment — Submitted filename: Reviewers Response.docx [file pone.0258863.s007.docx]

The Editor-in-Chief

PLosOne

Dear Editor-in0chief,

Rebuttal letter

Thank you for the review of our manuscript and for the opportunity to revise the manuscript. The manuscript has been reviewed based on the feedback from reviewer #2 and the Academic Editor.

The point-by-point response to the comments may be found in the table below.

| Academic Editor’s Comments | Table 1 should be modified to include the reference, number of participants and primary results | This has been done. You may refer to Table 1 for details. Also the redacted parts of the manuscript have been presented in relevant sections. | | |
| --- | --- | --- | --- | --- |
|  | It is unclear how the authors reach their conclusion that no intervention increases EID when several study designs appear to significantly increase EID. The authors should discuss how bias in study design can alter these results. | The conclusion that no intervention was effective to increase EID uptake despite some significant odds ratio was because “usual care” rather than the intervention was favored.  We have highlighted this in paragraph 1 of the discussion. We added “It is important to highlight some significant point estimates (odd ratio) with narrow confidence intervals that favored routine practices instead of the intervention. eHealth (1.35, 0.93 to 1.96) and health systems improvement (1.67, 1.35 to 2.06) interventions fall in this category. Other analyses showed that most interventions were not effective because of very wide confidence intervals that included 1; the line of no effect. The small event rates of uptake of EID and positive EID results at 4-8 weeks of life were responsible for the findings”.  Authors are of the opinion that bias in study design would have been important if there were effective interventions that increased EID uptake, especially if observational studies contributed data to the analysis. The results were more influenced by factors discussed in the preceding paragraph. | | |
| Reviewer 2  Overall Comments | This is an important study given the global prevalence of children living with HIV of which a significant proportion are not aware of their diagnosis and not currently on treatment. The authors attempt to address the critical opportunities to identify infants living with HIV early that are being missed by critically reviewing and analyzing current published interventions aimed to increase the uptake of early infant diagnosis (EID). The study provided a review of the current literature, highlighting 16 studies published between 2014 and 2019 that evaluated interventions involving eHealth services, service improvement projects, service integration programs, behavioral interventions, and male partner involvement. Additionally, authors conducted a meta-analysis of each of these different intervention types to identify interventions that increase EID of HIV at 4-8 weeks of age.  Overall, the systematic review and meta-analysis is well done. The major strengths of this manuscript are the focus on a population (HIV exposed infants) that has high rates of morbidity and mortality if undiagnosed and not linked to care early in life, as well as the extensive search and selection of studies. However, a few additional points to consider, including unclear meta-analysis methodology, additional information on initial studies (including in table 1), and further clarification of conclusions based on evidence presented. I would also consider minor revisions including the use of identity-first vs person-first language, revising for punctuation and grammar throughout, incorrect formatting of methods section, and lack of appendices 4-6 included in submission supplementary materials. | We appreciate the acknowledgement of the reviewer. All suggested points have been reviewed and may be found further in the tabulated responses. | | |
|  | Specific comments of Reviewer #2 | | Response to comment | Location in manuscript |
| 1. | Within the methods section, it is unclear how many authors reviewed results and extracted data. The authors state two authors reviewed them, but list three different people (lines 141, 146, and 153). Please clarify how many authors reviewed/extracted data | | Three review authors screened studies for inclusion and performed risk of bias assessment while two authors extracted data. For clarity, the relevant sections of the methods section now reads: “Three review authors screened eligible studies for inclusion. While half of the potentially eligible studies was screened by BO and LK, the other half was screened by BO and NM. Further, for each pair of reviewers, a third reviewer resolved discordant screening”.  “Two review authors (BO, LK) independently extracted data”, and  “Three review authors performed risk of bias assessment of included studies, with half of the risk of bias assessment performed by BO and LK, the other half by BO and NM ---” | Lines 139- 141  Line 145  Lines 151 – 153 |
| 2. | Additional concern with the methodology is regarding the meta-analysis. Specifically explain why you chose to conduct the meta-analysis by intervention type and define measures utilized, including the effect estimate. It is also not clear which studies you included in the meta-analysis. | | Meta-analysis was conducted by intervention type because the primary objective of the review was to assess the effectiveness of different interventions to increase IED uptake. Furthermore, two previous systematic reviews (Ambia et. al 2016 & Vrazo et. al 2018) combined all interventions in meta-analysis and concluded that they improved EID uptake. This makes it impossible for Program funders and managers to know the exact intervention that was effective. This is the justification for the conduct of meta-analysis based on interventions in this systematic review.  The effect estimate of the meta-analysis may be found in line 168 of the manuscript.  Because meta-analysis was presented in the results section, studies whose data were involved in meta-analyses were referenced in the relevant sections of the results. Each study ID is indicated in the analyses’ Forest plots. Please refer to line 199 to 323 in the results section. In addition, each Forest plot had the study ID of studies in each meta-analysis. |  |
| 3. | Between the methods and results section, the blank spaces between lines 190-201 need to be removed/reformatted. | | The blank spaces are because each section of the manuscript starts on a new page. The spaces have been deleted. |  |
| 4. | A concern within the results section is that it is unclear what is a summary of the individual study results versus what are the results of the author’s meta-analysis. The authors could consider presenting a summary of the results as reported by the primary study authors and then the results of their meta-analysis by each intervention sub-type.  Authors could also provide a table with the cumulative data that each meta-analysis is based on to better support their meta-analysis results. | | I am unsure of what Reviewer #2 meant as “summary of the individual study results”. The only place where narrative summary of data of included studies was presented is for Turnaround Time of EID results. The reasons for this were justified in the methods section, based on the recommendations of the Cochrane Handbook of Systematic Reviews. Other results were presented as either meta-analysis odds ratio (point estimate) and the 95% confidence intervals.  Per adventure Reviewer #2 was referring to the use of point estimates of odds ratios (OR) and their confidence intervals used for sensitivity analyses as in an example with SMS only interventions, this is usual for meta-analysis, especially when the authors need to highlight the results of the meta-analysis based on categories of eHealth interventions as was done in this systematic review. Also, because we did not combine data from different study designs, we presented the point estimate (OR) and 95% confidence intervals for each study. For example: A cluster-randomized study involving 690 HEIs has an effect size of 1.57 (1.08 to 2.30)^29^.  Each meta-analysis forest plot has cumulative data with study ID of included studies. Also, the revised Table 1 has all data of each included study. Authors prefer to present all data in more conventional formats to avoid undue duplication of data presentation. | Result section  Line 261  Kindly refer to the file that has the meta-analyses |
| 5. | An additional concern with the results is summary of information included in Tables 1 and 3. Authors should consider re-organizing Tables 1 and 3 to mirror results section (e.g., organized by intervention type with subheadings as opposed to study design). Additionally, Table 1 should provide additional useful information about each study, including number of participants and study results. Table 1 should also include references so that readers can easily refer to cited studies. Also it is unclear what the serial number in the tables are for and authors could consider removing these. | | Table 1 has been revised and serial number has been removed.  Studies have been re-arranged based on intervention type. The reference, and pre-specified outcome data and number of participants for each included study have been added to Table 1. This aligns also with the suggestion of the Academic Editor.  Appendix 3 (Characteristics of included studies) has been re-organized based on intervention types. | See Table 1 and Appendix 3 |
| 6. | Within the appendices, figure 1 arrowheads at the top of the PRISMA flow diagram are incorrect. Authors should fix the formatting of the arrows. | | The PRISMA flow diagram has been replaced with the 2020 version of the PRISMA flow diagram. | Figure 1 file |
| 7. | An additional concern is the authors conclusion within the discussion. It is unclear how authors came to the conclusion that there was no evidence that any of the evaluated interventions increased uptake of EID compared to usual care based on the evidence you present in the results section, including statistically significant effect estimates/OR’s showing what appears to be uptake in EID | | This has been addressed as one of the responses to the comments of the Academic Editor. |  |
| 8. | I am concerned with the authors use of identity-first language in throughout the manuscript. The authors should use person-first language throughout the manuscript. For example: “HIV infected infants” can be changed to “infants living with HIV.” | | HIV-infected infant(s) has been replaced with Infant(s) living with HIV in the Introduction and Discussion sections of the manuscript. Also, “HIV-positive women” has been replaced with “women living with HIV” in the Appendix 3.  HIV-exposed infants and “identification of HIV-infected infants” have been retained because the phrases are relevant to the early infant diagnosis (EID) of HIV infection. More so, HIV infection diagnosis is only known after EID results are positive. | The changes are all through the manuscript |
| 9. | Authors should revise for punctuation and grammar throughout the manuscript | | We have use Language Editing services for the revised manuscript | All through the manuscript. |
| 10. | Authors refer to appendices 4-6, however these were not provided in the supplementary materials. | | A big apology for the omission. Appendices 4-6 have been added as supplementary files. | Pages 13-19 of the Appendices’ file |

Warm regards,

Dr. B.O Okusanya

Corresponding Author
